# Marine n-3 Fatty Acids and Gene Expression in Peripheral Blood Mononuclear Cells

**DOI:** 10.1007/s12170-014-0412-7

**Published:** 2014-09-27

**Authors:** Stine M. Ulven, Mari C. Myhrstad, Kirsten B. Holven

**Affiliations:** 1 Department of Health, Nutrition and Management, Faculty of Health Sciences, Oslo and Akershus University College of Applied Sciences (HiOA), P.O. Box 4, St Olavs plass, 0130 Oslo, Norway; 2 Department of Nutrition, Institute for Basic Medical Sciences, University of Oslo, P.O. Box 1046, Blindern, 0317 Oslo, Norway

**Keywords:** Cardiovascular disease, Marine n-3 fatty acids, Dietary intervention study, Peripheral mononuclear cells, PBMCs, Inflammation, Lipid metabolism, Transcriptomics, Gene expression

## Abstract

Intake of marine n-3 fatty acids has been shown to have beneficial effects on cardiovascular disease. Gene expression analyses in peripheral blood mononuclear cells (PBMCs) are used to understand the underlying mechanisms of action of marine n-3 fatty acids. The aim of this review was to summarize the effects mediated by marine n-3 fatty acids on gene expression in PBMCs. A systematic literature search was conducted in PubMed in May 2014 and 14 papers were included. Targeted gene expression studies were reported in 9 papers and focused on genes involved in lipid metabolism and inflammation. Whole genome transcriptome analyses were conducted in 5 papers, and processes and pathways related to atherosclerotic plaque formation such as inflammation, oxidative stress response, cell cycle, cell adhesion, and apoptosis were modulated after fish oil supplementation. PBMC gene expression profiling has a potential to clarify further the molecular effects of fish oil consumption on human health.

## Introduction

Fish consumption reduces the risk of developing cardiovascular disease (CVD) and CVD mortality [1, 2]. Intervention trials with fish and fish oil containing the marine n-3 fatty acids eicosapentaenoic acid (EPA, 20:5 n-3) and docosahexaenoic (DHA, 22:6 n-3) have shown reduced total mortality and major coronary event including fatal and nonfatal MI [3–6]. However, recent meta-analyses and systematic reviews have shown that contradictory results exist regarding the beneficial effects of marine n-3 fatty acids on secondary prevention of coronary heart disease [7, 8]. The health beneficial effects of marine n-3 fatty acids are suggested to be mediated by reducing plasma triglycerides, reduced resting heart rate and blood pressure, and n-3 fatty acids may improve vascular function and immune response [9, 10]. Most organizations and national health councils recommend regular intake of fatty fish (1-2 servings per week of fatty fish) in order to prevent CVD. Specific dietary recommendations for the marine n-3 fatty acids are not widely established and what those levels ought to be for particular populations are unclear. 

Marine n-3 fatty acids regulate expression of genes involved in lipid metabolism and inflammation by acting as ligands for the peroxisomal proliferator-activated receptors (PPARs) [11]. In addition, fatty acids have the ability to reduce the activation of the transcription factor nuclear factor kappa B (NF-κB) probably by interference with the PPARs [12, 13] or the Toll-like receptors [14, 15]. Recently, the G protein-coupled receptor 120 (GPR120) has been characterized as n-3 fatty acid receptor/sensor involved in mediating the anti-inflammatory effects of n-3 fatty acids [16]. The ability of marine n-3 fatty acids to alter gene expression may account for their potential health beneficial effects. To further understand the underlying molecular mechanisms of inclusion of fish oil and the marine n-3 fatty acids in the human diet, the peripheral blood mononuclear cells (PBMCs) may, therefore, provide a model system for studying gene expression of mediators involved in the early development of atherosclerosis [17, 18]. The PBMCs, which include monocytes and lymphocytes, are exposed to many of the same environmental factors as metabolic tissues and the arterial wall. PBMCs are also readily available, which make them suitable for studying gene expression in dietary intervention studies.

The aim of this review is to summarize the effects of marine n-3 fatty acids on gene expression in PBMCs and discuss the findings in relation to human health.

### Literature Search

**Fig. 1:**
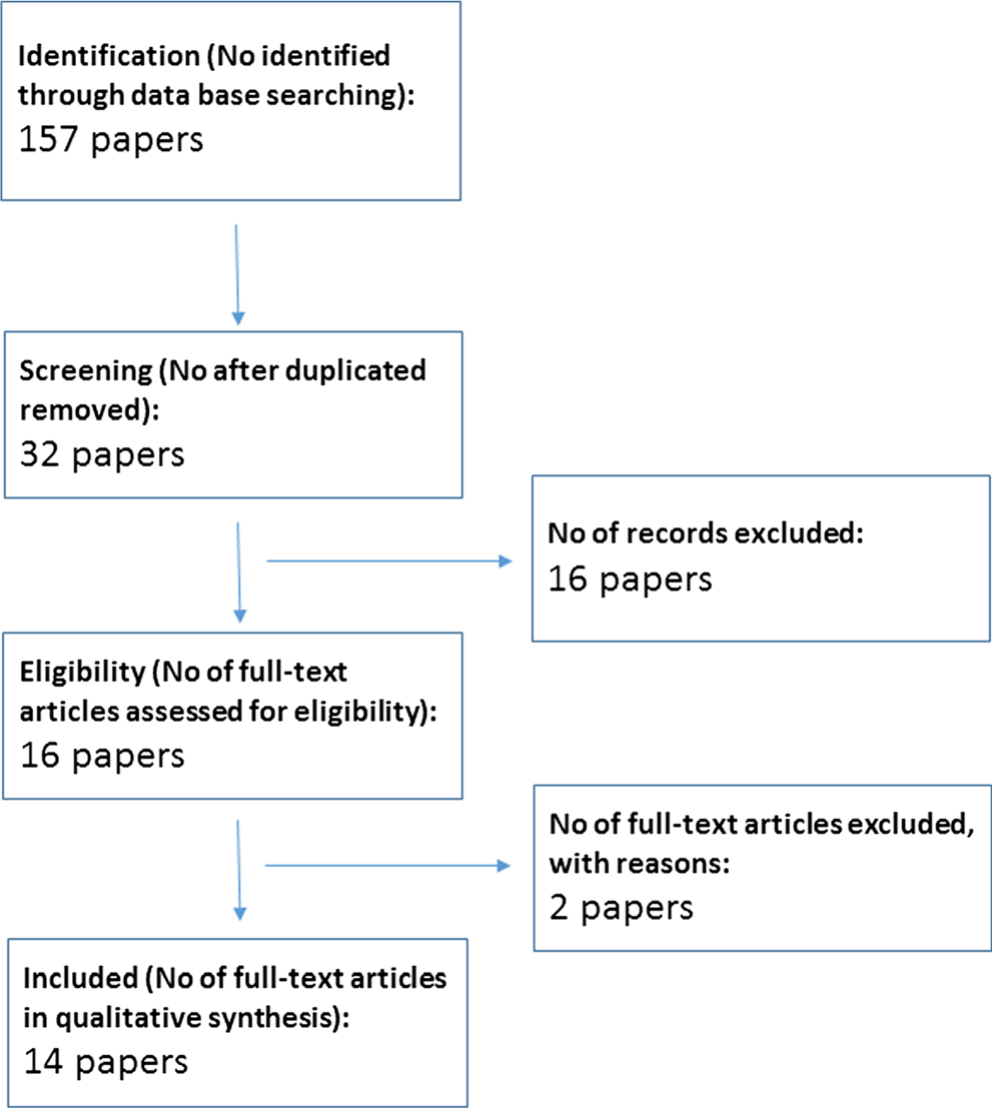
A flow chart of the PubMed search and selection of papers

A systematic literature search was conducted in Pubmed in May 2014 using the following terms: “(fish oil and gene expression and peripheral blood mononuclear cells), (fish oil and transcriptome and peripheral blood mononuclear cells), (fish oil and mRNA expression and peripheral blood mononuclear cells), (n-3 fatty acids and gene expression and peripheral blood mononuclear cells), (n-3 fatty acids and transcriptome and peripheral blood mononuclear cells), (n-3 fatty acids and mRNA expression and peripheral blood mononuclear cells), (fish oil and gene expression and mononuclear cells), (fish oil and transcriptome and mononuclear cells), (fish oil and mRNA expression and mononuclear cells), (n-3 fatty acids and gene expression and mononuclear cells), (n-3 fatty acids and transcriptome and mononuclear cells), (n-3 fatty acids and mRNA expression and mononuclear cells)”. In total 157 instances of articles were identified, but after removing duplicates, 32 papers were screened by reading abstracts (Fig. 1). In total 16 papers were excluded based on the following criteria of not be a human study, not using fish oil or marine n-3 fatty acids, no gene expression data, not an original paper (Fig. 1). After reading 16 full-text papers, 2 more papers were excluded because there was no effect of fish oil or n-3 fatty acids on PBMC gene expression was shown in these papers (Fig. 1). In total, 14 original papers were included in the review.

## Fish Oil Intervention Studies and Targeted Gene Expression Studies in PBMCs

Table 1 summarizes the effect of marine n-3 fatty acids from 9 targeted gene expression studies in PBMCs. In total, 7 fish oil intervention studies have used PBMC gene expression data of which one was a postprandial study (Table 1). The duration of the studies ranged from 4–12 weeks. Kaminski et al studied for the first time in 1993 the effect of marine n-3 fatty acids on platelet-derived growth factor (PDGF) gene expression in human mononuclear cells (MNC) [19]. In a controlled randomized, observer-blinded study with 14 healthy males, intake of 7 g/d of fish oil concentrate (54 % EPA and 28.7 % DHA) reduced the mRNA level of PDGF-A and B after both 1 and 6 weeks of intervention (6 out of 7 subjects) [19]. In the control, group, receiving no supplement, the expression level of PDGF-A remained unaltered in 6 out of 7 subjects. To further examine other growth factors, known to be involved in proliferation of endothelial cells, Jendraschak et al analyzed the mRNA expression level of platelet-derived endothelial cell growth factor (PD-ECGF), insulin-like growth factor (IGF-1A, 1B), and transforming growth factor-β1 (TGF-β1) [20]. The expression level of PD-ECGF was constant, whereas gene expression of TGF-β1, IGF-1A, and IGF-1B were varying over time and among subjects, independently of each other [20]. Baumann et al performed a 4-week randomized, investigator-blinded intervention trial with 7 g/d of fish oil concentrate (41.4 % EPA and 23.6 % DHA) (*n* = 7), 7 g/d of corn oil concentrate (50.1 % linoleic acid) (*n* = 7), or 7 g/d of olive oil concentrate (60.5 % oleic acid) (*n* = 7) vs no dietary intervention (*n* = 7) [21]. The MNC gene expression levels of PDGF-A, PDGF-B, and monocyte chemoattractant protein-1 (MCP-1), also called chemokine (C-C motif) ligand 2 (CCL2), were reduced in the fish oil group in accordance with the data from Kaminski et al [19]. Interleukin-10 (IL-10) and heparin-bound epidermal growth factor (HB-EGF) mRNA levels were not changed by fish oil supplementation [21]. The MNC mRNA levels of PDGF-A, PDGF-B, MCP-1, IL-10, and HB-EGF were not altered in the n-6 and n-9 fatty acid groups [21]. PDGF is a chemoattractant for neutrophils, monocytes, fibroblasts, and smooth muscle cells. Proliferation of vascular endothelial and intimal smooth muscle cells, characteristic for atherosclerotic lesions, may be initiated and maintained by PDGF-like protein secreted by monocytes, and fish oil consumption seems to regulate the expression of this gene in PBMCs, which may have a health beneficial effect on development of atherosclerosis.

**Table 1:** PBMC target gene expression in marine n-3 fatty acid intervention studies ^a^ Number of subjects included in mRNA analyses

Study	Study design	Intervention	Intervention duration (wk)	Subjects ^a^ (n, age)	Genes	Upregulated	Downregulated	No effect
Cruz-Teno et al, 2012	Randomized dietary intervention study	Four diets; High saturated fat (HSFA) or high monounsaturated fatty acids (HMUFA) or low-fat, high complex carbohydrate supplemented with n-3 (LFHCC n-3) or high complex carbohydrate (LFHCC) (placebo)	12 and 4 h postprandial challenge	75 subjects with metabolic syndrome	IκB-α, TNFα, MMP9, IL-6, MCP-1, p65, MIF	Fasting: higher IκB-α mRNA after LFHCC n-3 compared with HSFA and HMUFA. Postprandial: 12 wk consumption of HMUFA increased postprandial (fat load; 4 h) gene expression of IκB-α compared with HSFA and LFHCC n-3 groups.		No difference in postprandial PBMC p65, MIF, and MCP-1, mRNA between the 4 diets.
Telle-Hansen et al, 2012	Randomized controlled dietary intervention study	Three groups; 150 g of cod or 150 g salmon, or potato (control) daily	15 d	30 Healthy male and female, 20–40 y	SCD-1, FAS	The mRNA level of FAS significantly increased within the salmon group.		No effect on SCD-1 or FAS after intervention with cod or salmon compared with potato.
Myhrstad et al, 2011	Crossover study, fixed-order meals.	Three test meals (chocolate cakes): (1) coconut cake (43 E% from SFA); (2) Linseed cake (14 E% from ALA); and (3) cod liver oil cake (5 E% from ALA, 5 E% from EPA and 3 E% from DHA). Ex vivo PBMC stimulation with ALA and EPA	Postprandial	14 Healthy young women, 22–25 y (IQR)	IL-8, IL-1β, IL-6, PPARγ and CPT1a	Intake of cod liver oil cake: significant increase in the mRNA level of IL-8 after 6 h compared with fasting. Significant increase in the mRNA level of CPT1A after 6 h compared with 3 h. Ex vivo stimulation: EPA increased gene expression of IL-8 and CPT1A compared with unstimulated cells.		No significant difference in mRNA level of IL-1β, IL-6, and PPARγ compared with fasting or between the different cakes. No difference in mRNA levels of CPT1A between the meals. Ex vivo stimulation: EPA had no effect on IL-1β.
Radler et al, 2011	Placebo-controlled double blind	Two groups: low-fat yogurt (125 g) containing grapeseeds (81 mg polyphenols), fish oil (100 mg n-3 PUFA), phospholipids (400 mg), L-carnitine (1 g) vitamin C (60 mg), and vitamin E (10 mg) (PPC) or low-fat yogurt containing only vitamin C (60 mg) and vitamin E (10 mg)	12	22 moderately hyperlipidemic obese subjects, 53.9 y ± 10.9 and 20 matching participants, 53.8 y ± 10.4	PPARα, CPT1A, CPT1B, CrAT, OCTN2	The PPC administration increased mRNA levels of PPARα, CPT1A, CPT1B, CrAT, and OCTN2 . No effect on these genes in control group before and after intervention.		
Weaver et al, 2009	“Crossover”: 4 wk dietary supplements and 2 wk washout. Not randomized	One group: dietary supplement; fish oil (775 mg EPA/d), and Borage oil (831 mg GLA/d)	4	27 healthy volunteers	PI3K, Akt, NFκB, IL-1β, IL-10, IL-23, IL-5, IL-17, TNFα		Significant decrease in PBMC gene expression of PI3Kα, PI3Kγ. Cytokines; IL-10 and IL-23 mRNA were decreased after supplementation. Borderline significant decrease for IL-1β (*P* = 0.09), IL-5 (*P* = 0.06), and IL-17 (*P* = 0.09) after supplementation.	No difference in gene expression of Akt, NFκB, PI3Kδ, and PI3Kβ, IL-12, TNFα, and IL-6 after supplementation.
de Mello et al, 2009	Randomized dietary intervention study	Three groups: fatty or lean fish (4 meals per wk) or control (chicken)	8	27 subjects with myocardial infarction or unstable ischemic attack during the last 3 mo, >70 y	TNF, IL-1β, IL-6, CCL2, CCL5, ICAM1, VCAM1, E-selectin, and P-selectin.			No significant differences in both fish groups were observed.
Baumann et al, 1999	Randomized controlled study, investigator-blinded	Four groups: fish oil 7 g/d, corn oil 7 d/d or olive oil 7 d/d or no dietary intervention	4	28 healthy volunteers, 20–38 y	IL-10, PDGF-A, PDGF-B, MCP-1, and HB-EGF.		Reduced expression of PDGF-A, PDGF-B, and MCP-1 (25 %, 31 %, and 40 %, respectively), in MNC after fish oil consumption.	No effect of fish oil on mRNA expression of IL-10 and HB-EGF.
Kaminski et al, 1993	Controlled, randomized observer-blinded study	Two groups; fish oil group; 7 g/d fish oil concentrate (54.7 % EPA, 28.7 % DHA, 5.4 % C22:5, n-3; 2.4 % C20:4, n-3; 2.4 % C18:4, n-3; 2.1 % C21:5, n-3; and control group.	6	14 healthy volunteers, 28.9 ± 3.5 y	PDGF-A, PDGF-B		Reduced PBMC gene expression of PDGF-A and PDGF-B after fish oil consumption.	
Jendraschak et al, 1993	Controlled, randomized observer-blinded study	Two groups; fish oil group; 7 g/d fish oil concentrate (54.7 % EPA, 28.7 % DHA, 5.4 % C22:5, n-3; 2.4 % C20:4, n-3; 2.4 % C18:4, n-3; 2.1 % C21:5, n-3 and control group	6	14 healthy volunteers, 28.9 ± 3.5 y	PD-ECGF, TGF-β1, IGF-1A, IGF-1B.			No effect of fish oil on mRNA expression of PD-ECGF, TGF-b1, IGF-1A, IGF-1B.

*ALA* alpha linolenic acid, *CCL* chemokine (C-C motif) ligand, 2, 5 *CPT1A* carnitine palmitoyltransferase-1A, *CrAT* carnitine acetyl-transferase, *EPA* eicosapentaenoic acid, *FAS* fatty acid synthase, *GLA* gamma-linolenic acid, *HB-EGF* heparin-bound epidermal growth factor, *ICAM-1* intercellular cell adhesion molecule-1, *IGF-1A* insulin-like growth factor 1A, *IKB* inhibitor of kappa B, *IL* Interleukin, 1b, 5, 6, 10,12, 17, 23, *NFκB* nuclear factor kappa-light-chain-enhancer of activated B cells, *MCP-1* monocyte chemoattractant protein, *MIF* macrophage migration inhibitory factor, *MMP-9* matrix metalloproteinase 9, *OCTN2* organic cation transporter P65 nuclear factor NF-kappa-B p65 subunit, *PD-ECGF* platelet-derived endothelial cell growth factor, *PDGF* platelet derived growth factor, *PUFA* polyunsaturated fatty acids, *PI3k* phosphatidylinositol 3-kinase, β,δ. PPARγ, *PPAR*γ peroxisomal proliferator-activated receptor γ, *SFA* saturated fatty acids, *SCD-1* stearoyl CoA desaturase, *TGF-β1* transforming growth factor-β1, *TNF*α tumor necrosis factor alpha, *VCAM-1* vascular cell adhesion molecule -1

Weaver et al performed a dietary “cross-over” study in which 27 healthy adults were given a controlled background diet together with fish oil (775 mg EPA/d) and borage oil (831 mg γ-linolenic acid (GLA18:3, n-6)/d) for 4 weeks followed by a 2- week wash-out period [22]. Supplementation of fish oil and borage oil induced a decrease in the expression of phosphatidylinositol 3-kinase (PI3K) α and PIK3Kγ [22]. The expression of PI3Kβ, PI3Kδ, protein kinase B, also known as Akt and NF-kB was not significantly altered during supplementation [22]. PI3Kα and PIK3Kγ play an important roles in eicosanoid formation and in cell growth, survival, and inflammation by modulating Akt and NF-κB signaling, which in turn influence the production of a variety of signaling molecules such as cytokines. Furthermore, 8 cytokines known to play an important role in the inflammatory response were measured in the same study. The mRNA expression level of interleukin- (IL)-1β, IL-10, and IL-23 were significantly decreased after supplementation with fish oil and borage oil [22]. In addition, the expression of IL-5 and IL-17 showed trends toward decreased expression after supplementation, whereas the expression of TNF-α and IL-6 were not changed [22]. This study shows that 4-week supplementation with fish oil and borage oil reduces the expression of PI3Kα and PIK3Kγ, which are early steps in cellular signal transduction, as well as reduce expression of several important downstream genes such as different interleukins. If the effect is mediated via altered formation of inflammatory eicosanoids or a direct effect on gene regulation is not known. It cannot be ruled out, that the effects may also have been caused by a change in n-6 and n-3 PUFA content and not only mediated by the marine n-3 fatty acids.

In a placebo-controlled double blind study with 22 moderately hyperlipidemic obese humans consuming low-fat yogurt enriched with a combination of low-dose PUFAs from fish oil (100 mg n-3 fatty acids), polyphenols and L-carnitine (PPC) twice a day for 12 weeks, the expression of genes involved in lipid metabolism were compared with 20 matching participants ingesting low-fat yogurt [23]. The level of plasma free fatty acids and triglycerides were significantly reduced in the PPC group. In addition, the PBMC gene expression of PPARα, carnitine palmitoyltransferase-1, CPT1A, and CPT1B, carnitine acetyl-transferase (CrAT) and organic cation transporter 2 (OCTN2) was significantly increased in the PPC group [23]. No change was seen in the control group [23]. This study showed that a reduction in plasma free fatty acids and triglycerides coincided with increased PBMC mRNA expression level of genes encoding proteins involved in fatty acid oxidation among subjects consuming PCC. Because CPT1 is regulating the capacity for mitochondrial fatty acid oxidation, the increase in mRNA expression of CPT1 may stimulate oxidation and result in the subsequent observed decrease in free fatty acids. The observed increase in the mRNA level of PPARα may also partly explain the increased expression of CPT1, CPT2, and OCTN2 [24]. However, the effects seen in this study may also be related to intake of carnitine and not only mediated by the marine n-3 fatty acids.

Cruz-Teno et al performed a randomized dietary intervention study with 75 subjects with metabolic syndrome allocated to 1 of 4 diets; high saturated fatty acids (HSFA); high monounsaturated fatty acids (HMUFA) and 2 low-fat, high complex carbohydrate (LFHCC) diets, supplemented with n-3 fatty acids (LFHCC n-3) or placebo (LFHCC), for 12 weeks, followed by a postprandial challenge [25•]. In the HMUFA diet group and 4 hours after the fat overload, a significant postprandial increase in the mRNA expression level of nuclear factor of kappa light polypeptide gene enhancer in B-cells inhibitor, alpha (IkB)- α compared with HSFA and LFHCC n-3 diets was observed [25•]. In contrast, at fasting levels, after 12 weeks consumption of LFHCC n-3 diet, the expression level of IKB-α mRNA was higher compared with HSFA and HMUFA diets. Intake of the 4 different fat load breakfasts induced an increase in the mRNA expression of TNFα, metalloproteinase 9 (MMP-9), and IL-6 independently of the diet consumed, reflecting an acute inflammatory response during the postprandial period. No changes in NF-κB p65 subunit (p65), macrophage migration inhibitory factor (MIF) and MCP-1 mRNA levels were observed after intake of any of the 4 different diets [25•]. The postprandial plasma protein concentration of MCP-1 was reduced after intake of HMUFA and LFHCC n-3 diets compared with HSFA diet. After the fat load, an increase in the postprandial plasma protein levels of IL-6 independently of diet consumed was observed. No effects were found by diet on plasma protein level of TNFα [25•].

We have also performed a postprandial study where 3 different test meals were consumed by 14 healthy women in a fixed order with 14 days wash-out between each test meal [26•]. The test meals consisted of 3 different 150 g chocolate cakes enriched with coconut fat [43 % energy as saturated fat and 1 % energy as alpha-linolenic acid (ALA)], linseed oil (14 % energy as ALA and 30 % energy as saturated fat), and cod liver oil (5 % energy as EPA and DHA and 5 % energy as ALA in addition to 31 % energy as saturated fat). At 3 hours after intake of the test meal, the plasma triglycerides increased in all groups, whereas no significant differences in triglycerides were observed after 6 hours compared with baseline in any of the groups. The mRNA expression level of IL-8 was significantly upregulated after intake of the cod liver oil cake at 6 hours compared with fasting level. This increase was significantly different from the effect observed after intake of linseed oil cake. No increase in plasma protein level of IL-8 was observed for any of the test meals. The mRNA level of CPT1A was also significantly increased 6 hours after intake of cod liver oil cake compared with 3 hours. In addition, ex vivo cultured PBMCs were incubated for 24 hours with 60 μM of EPA. Incubation with EPA significantly increased the mRNA expression level of IL-8 and CPT1A compared with unstimulated cells [26•].

## Fish Intervention Studies and Targeted Gene Expression Studies in PBMCs

Of the total 9 papers on targeted gene expression analysis, 2 dietary intervention studies with fatty or lean fish have been reported. De Mello et al showed in a 8-week randomized study with 3 groups, fatty fish diet (*n* = 11, 0.89 g EPA+DHA/d), lean fish diet (*n* = 11, 0.43 g EPA+DHA/d) and control diet (*n* = 6, 0.18 g EPA+DHA/d) among patients with coronary heart disease, that 4 portions of fish per week for 8 weeks did not differently change the gene expression of TNF, IL-1β, IL-6, chemokine (C-C motif) ligand 2 (CCL-2), CCL-5, intercellular cell adhesion molecule-1 (ICAM1), vascular cell adhesion molecule -1 (VCAM1), and E- and P-selectin during the intervention in neither the fatty fish or the lean fish group [27]. We have also shown in a 15-day randomized study with 3 groups, fatty fish diet (*n* = 11, 3.1 g EPA+DHA/d), lean fish diet (*n* = 9, 0.13 g EPA+DHA/d), and control diet (*n* = 10, 0 g EPA+DHA/d) among healthy adults, that 150 g of fish per day did not change the gene expression of stearoyl-CoA desaturase-1 (SCD-1) and fatty acid synthase (FAS) in PBMCs compared with the control group [28]. We did, however, observe an increase in FAS mRNA level within the salmon group. The plasma triglyceride levels were significantly decreased in both fish groups compared with the control group, and HDL-cholesterol was significantly increased after intake of salmon compared with the control group. Total-cholesterol and LDL-cholesterol were not changed between or within any of the groups during the intervention [28].

Overall, few targeted gene expression studies have been performed and a limited number of genes related to lipid metabolism (such as PPARα, CPT1, FAS, and SCD-1) have been examined. Most of the effects seen are related to genes involved in immune function. In many of these studies the effect has been studied within the group receiving fish oil and not in comparison with the control group.

### Fish Oil Interventions and Whole Genome Transcriptomics in PBMCs

PBMC transcriptome analyses in human intervention studies with fish oil were reported in 5 papers and are summarized in Table 2. Three of the studies were double-blinded randomized placebo-controlled studies and all used fish oil (1.6 g/d EPA+DHA/d-3 g/d EPA+DHA) and included healthy adults or elderly subjects, subjects with Alzheimer disease and obese insulin resistant subjects. The duration of the studies ranged from 6–26 weeks (Table 2).

**Table 2:** PBMC transcriptome analyses in marine n-3 fatty acid intervention studies

Study	Study Design	Intervention^b^	Intervention duration (wk)	Subjects^a^ (n, age)	Microarray platform	Regulated genes	Regulated processes, pathways/networks	Biomarkers related to CVD
Myhrstad et al, 2014	Double-blinded, randomized, placebo-controlled study.	8 g/d fish oil (1.6 g EPA + DHA/d) or 8 g/d HOSO (control)	7	36 healthy subjects, 18–50 y, M/F	Illumina HumanHT-12 v4	Between groups: 470 gene transcripts (*P* < 0.05)	Between groups: biological processes and pathways related to cell cycle, DNA package/chromosome organization, ER stress response, apoptosis, and survival. ↑	Not included
Rudkowska et al, 2012	Intervention study, not placebo-controlled.	5 g/d fish oil (3 g EPA + DHA/d)	6	29 healthy subject, 12 M (mean age 33.5 y ); 17 F (mean age 34.4 y)	Illumina HumanHT-6 v3	Pre- to postintervention: M + F: 170 gene transcripts (*P* < 0.05) M: 610 gene transcripts (*P* < 0.05) F: 250 gene transcripts (*P* < 0.05)	Within group: changes in pathways related to NrF2 oxidative stress response, PPARγ activation of gene regulation, HIF signaling, NF-*κ*B signaling. Differences in response between M and F.	M + F: TG ↓ TC, LDL, IL-6, TNFα, CRP ↔
Vedin et al, 2012	Double-blind, randomized placebo-controlled study.	1.7 g DHA + 0.6 g EPA/d (EPAX 1050TG) or 1 g/d Corn oil with LA (control)	24	16 Alzheimer disease (AD) subjects	Human Genome Focus Array (Affymetrix)	Between groups: 5 gene transcripts (*P* < 0.05), CD63, RHOB, LOC399491, ZNF24, ANAPC5 Within n-3 group: 19 gene transcripts (FDR <10 %)	Within n-3 group: hanges in gene transcripts related to inflammation, ubiquitination, and neurodegeneration.	Not included
Rudkowska et al, 2011	Randomized controlled study, crossover design.	Fish oil: 1.8 g EPA + DHA/d or fish oil + fish gelatin (1.8 g EPA + DHA/d and 25 % of daily protein intake).	8	16 obese, insulin resistant subjects, M and F	Illumina HumanHT-6 v3	Within FO group: 805 gene transcripts Within FO + FG group: 184 gene transcripts (Fold change <0.8 or >1.2 and *P* < 0.05) Overlap: 3 genes FADS1, EFAR3, and EDA	Within fish oil and fish oil + fish gelatin group: similar changes in the pathway related to PPARγ. Changes in pathways related to HIF signaling, NF-*κ*B, oxidative stress response via Nrf2 in both groups but to different extent.	TG ↓ TC, HDL, LDL, IL-6, TNFα, CRP ↔
Bouwens et al, 2009	Double-blind, randomized, placebo-controlled study.	1.8 g/d EPA + DHA or HOSO (control)	26	48 elderly healthy subjects	Human whole-genome NuGO Gene Chip arrays (Affymetrix)	Between groups: not shown within n-3 group: 1040 gene transcripts (*P* < 0.05). Within HOSO group: 298	Within n-3 group: pathways related to NF-*κ*B-signaling, eicosanoid synthesis, scavenger receptor activity, adipogenesis, and hypoxia signaling. ↓ Pathways related to cell cycle. ↑	FFA and TG↓, CRP ↔ ^c^

^a^ Number of subjects included in transcriptome analyses

^b^Intervention groups included in transcriptome analyses

^c^No changes between the groups included in the transcriptome analyses

*ANAPC5* Anaphase promoting complex subunit 5, *CRP* C reactive protein, *EDA* ectodysplasin, *EFAR3* free fatty acid receptor 3, *F* female, *FADS1* fatty acid desaturase 1, *FFA* free fatty acids, *HDL* High density lipoprotein, *HIF* hypoxia-inducible factor, *HOSO* High oleic sunflower oil, *IL-6* Interleukin-6, *LDL* low density lipoprotein, *LOC399491* LOC 3999491 protein, *M* male, *NF-κB* nuclear factor kappa B, NrF2 nuclear factor (erythroid-derived 2) like 2, *PPARalpha* peroxisomal proliferator-activated receptor a, *RHOB* Ras homolog gene family, member B, *TC* total cholesterol, *TG* triglyceride, *TNF*α Tumor necrosis factor alpha, *ZNF24* Zinc finger protein 24

Bouwens et al [29] first reported the effect of fish oil supplementation on whole genome gene expression profiles in human PBMCs in 2009. A double-blinded randomized intervention trial in an elderly population was performed. The triglyceride levels were significantly reduced in the fish oil group compared to the high oleic sunflower oil (HOSO, control) group, but no change in CRP level was observed in any of the groups. Within the fish oil group consuming 1.8 g/d EPA+DHA (*n* = 23), 1040 gene transcripts were regulated after 26 weeks of intervention. Within the high oleic sunflower oil (HOSO, control) group (*n* = 25), 298 genes were regulated after 26 weeks of intervention. In total 140 genes were overlapping between the groups, and the direction of change was the same in both groups. Pathways analysis (using GenMAPP) showed that fish oil supplementation for 26 weeks significantly decreased the expression of genes involved in inflammatory pathways, such as eicosanoid synthesis, interleukin signaling, and MAP kinase signaling. Fish oil supplementation also significantly decreased expression of genes related to atherosclerotic processes, such as cell adhesion, scavenger receptor activity, and adipogenesis. Other pathway analysis and gene set enrichment analyses (GSEA analysis) showed a decrease in similar inflammatory signaling pathways as described above in the fish oil group. In addition, these analyses also showed that genes involved in nuclear transcription factor kB (NF-kB) and Toll-like receptor signaling were decreased in the fish oil group. A decrease in oxidative stress, cell adhesion, cell cycle, PPAR and LXR/RXR activation, and hypoxia signaling was also observed in the fish oil group. In the HOSO group, pathways analysis showed a decreased expression of genes involved in inflammation and cell adhesion [29].

Vedin et al showed in a 24-week double-blinded randomized, placebo-controlled fish oil study (2.3 g/d EPA+DHA) among 16 subjects with Alzheimer disease that 19 genes (with a false discovery rate of 10 %) were changed in the fish oil group [30•]. In total, 9 genes were upregulated by the fish oil supplementation, and of these, 1 gene was also upregulated in the control group (corn oil), and 1 gene was significantly different in fold change between the 2 groups (CD63 molecule). In total, 10 genes were downregulated by the fish oil supplementation, and of these, 4 genes were significantly different in fold change between the groups [Ras homolog gene family, member B (RHOB), LOC 3999491 protein (LOC399491), zinc finger protein 24 (ZNF24), and Anaphase promoting complex subunit 5 (ANAPC5)]. Many of the modulated genes are involved in inflammation regulation, and neurodegeneration and in ubiquitination processes [30•].

Rudkowska et al showed in a 8 weeks randomized controlled crossover study with fish oil (1.8 g/d EPA+DHA) and fish oil plus fish gelatin (+FG) with 16 obese insulin-resistant subjects that 805 genes were regulated in the fish oil group [31]. A decrease in fasting triglyceridesin both groups, but no changes in total cholesterol, LDL-cholesterol, HDL-cholesterol, and circulating inflammatory markers such as CRP, IL-6, and TNFα were observed. Among the 805 genes, 65 % (524) were downregulated and 35 % (281) were upregulated. In the fish oil plus fish gelatin group, 184 genes were regulated after an 8-week intervention (59 % (109) downregulated and 41 % (75) were upregulated). Of these genes, only 3 genes were overlapping in both supplementation periods [fatty acid desaturase 1 (FADS1), free fatty acid receptor 3 (EFAR3), and ectodysplasin (EDA)]. Pathways analysis (Ingenuity) showed that oxidative stress response mediated by nuclear factor (erythroid-derived 2) like 2 (NrF2), PPARα, hypoxia-inducible factor (HIF) and NF-κB signaling pathways were changed in both groups during the intervention but to different extents [31]. Rudkowska et al have also investigated the PBMC gene expression changes following fish oil supplementation (3 g/d EPA+DHA) for 6 weeks among 29 healthy adults (12 male and 17 females) [32]. The study was not a randomized study and was without a control group. They found that 170 genes were regulated after a 6-week intervention (47 % (80) downregulated and 53 % (90) upregulated). In male subjects, 610 genes were differently expressed (46 % (283) down-regulated and 54 % (327) upregulated) by fish oil supplementation. In females, only 250 genes were differently expressed (49 % (122) downregulated and 51 % (128) upregulated) by fish oil supplementation. Of these genes, only 9 genes were overlapping. Pathways analysis (Ingenuity) showed that oxidative stress response genes mediated by NrF2, PPARα, HIF and NF-κB signaling pathway were changed among males and females but to different extend. A decrease in fasting plasma triglycerides among males and females was observed, however, no other changes in total cholesterol, LDL-cholesterol, and circulating inflammatory markers such as CRP, IL-6, TNFα was observed [32].

In a 7-week double-blinded randomized controlled fish oil study (1.6 g/d EPA+DHA) with healthy adults, we have recently observed that 470 genes (*P* < 0.05) were differently expressed between the fish oil group and the HOSO group [33••]. Two hundred-and thirty-six genes were upregulated and 234 were downregulated in the fish oil group compared with the HOSO group. Among the upregulated genes, several biological processes were enriched such as cell cycle and DNA packing and chromosome organization. We found that 11 genes were significantly changed in the fish oil group (FDR q-value <10 %), and none in the HOSO group. Gene set enrichment analysis (GSEA) showed that in particular cell cycle, apoptosis, immune response, protein folding and maturation and DNA damage were significantly regulated in the fish oil group compared with the HOSO group. Moreover, Gene transcripts with common motifs for 35 known transcription factors including E2F, TP53, HIF, and ATF4 were upregulated after intake of fish oil compared with HOSO [33••].

Overall, 5 papers have reported using whole genome transcriptomics in fish oil intervention studies. Annotation and pathways analysis showed that fish oil supplementation modulated the expression of genes involved in inflammatory pathways, such as eicosanoid synthesis, interleukin signaling, and MAP kinase signaling, oxidative stress response, cell cycle, cell adhesion, apoptosis, scavenger receptor activity, adipogenesis, protein folding, maturation, and DNA damage [29–32, 33••]. Many of these are anti-atherogenic and anti-inflammatory genes, which play a role in several processes involved in atherosclerotic plaque formation. PBMCs, consisting of monocytes and lymphocytes are cells central in these processes including adhesion, infiltration, and foam cell formation, and, therefore, it seems like whole genome transcriptomics can be used to identify novel pathways modulated by intake of fish oil. Data from fish oil supplementation studies further indicates that these effects may be mediated via many transcription factors and signaling pathways, such as NF-κB, Toll-like receptor signaling, PPAR and LXR/RXR activation and hypoxia signaling [29, 33••]. The studies summarized in this paper have different duration, doses and study subjects, but overall they show that fish oil supplementation may influence several biological pathways and processes via different transcription factors. The discrepancy between the studies by Bouwens et al [29] and Rudkowska et al [31, 32] on PPARα activation, may be caused by differences in the duration of the studies or the age and genotype of the study subjects. Because it has been shown that the response of fish oil on triglycerides depends on sex and genotype [34], genetic differences may cause the variation in gene expression responses observed in these intervention studies. Another explanation could be that changes in gene expression are reflecting the response of PBMCs to EPA-DHA-induced systemic adaptations in the body and may, therefore, not be a direct effect of EPA+DHA on mononuclear cells. The major limitation in these transcriptomic studies is that most of the studies (4 out of 5) analyzed the within group changes, rather than between group differences.

## Conclusions

PBMC gene expression analysis in human dietary intervention studies with fish oil or fatty fish can be a powerful tool to understand the underlying molecular mechanisms explaining the association between high intakes of marine n-3 fatty acids and effects on morbidity and mortality from CVD. These studies show the potential of PBMC gene expression profiling to investigate the effects of nutrition on human health. Gene expression profiling seems to be more sensitive to fish oil interventions than the traditional biochemical parameters measured in circulation and enable us to study gene expression of mediators involved in the early development of atherosclerosis. Future studies should integrate gene expression patterns with biochemical parameters to determine the precise mechanisms of action of EPA and DHA in humans. More emphasis should also be on examining the gene expression profiles in PBMCs in responders versus non-responders in order to understand the influence of genetic factors on the effect of fish oil on lipids and inflammatory markers.  
